# Molecular Survey of Bacterial Zoonotic Agents in Bats from the Country of Georgia (Caucasus)

**DOI:** 10.1371/journal.pone.0171175

**Published:** 2017-01-27

**Authors:** Ying Bai, Lela Urushadze, Lynn Osikowicz, Clifton McKee, Ivan Kuzmin, Andrei Kandaurov, Giorgi Babuadze, Ioseb Natradze, Paata Imnadze, Michael Kosoy

**Affiliations:** 1 Division of Vector-Borne Disease, Centers for Disease Control and Prevention, Fort Collins, Colorado, United States of America; 2 National Center for Disease Control and Public Health, Tbilisi, Republic of Georgia; 3 Institute of Chemical Biology, Ilia State University, Tbilisi, Republic of Georgia; 4 Department of Biology, Colorado State University, Fort Collins, Colorado, United States of America; 5 Department of Pathology, University of Texas Medical Branch, Galveston, Texas, United States of America; 6 Institute of Zoology, Ilia State University, Tbilisi, Republic of Georgia; University of Pretoria, SOUTH AFRICA

## Abstract

Bats are important reservoirs for many zoonotic pathogens. However, no surveys of bacterial pathogens in bats have been performed in the Caucasus region. To understand the occurrence and distribution of bacterial infections in these mammals, 218 bats belonging to eight species collected from four regions of Georgia were examined for *Bartonella*, *Brucella*, *Leptospira*, and *Yersinia* using molecular approaches. *Bartonella* DNA was detected in 77 (35%) bats from all eight species and was distributed in all four regions. The prevalence ranged 6–50% per bat species. The *Bartonella* DNA represented 25 unique genetic variants that clustered into 21 lineages. *Brucella* DNA was detected in two *Miniopterus schreibersii* bats and in two *Myotis blythii* bats, all of which were from Imereti (west-central region). *Leptospira* DNA was detected in 25 (13%) bats that included four *M*. *schreibersii* bats and 21 *M*. *blythii* bats collected from two regions. The *Leptospira* sequences represented five genetic variants with one of them being closely related to the zoonotic pathogen *L*. *interrogans* (98.6% genetic identity). No *Yersinia* DNA was detected in the bats. Mixed infections were observed in several cases. One *M*. *blythii* bat and one *M*. *schreibersii* bat were co-infected with *Bartonella*, *Brucella*, and *Leptospira*; one *M*. *blythii* bat and one *M*. *schreibersii* bat were co-infected with *Bartonella* and *Brucella*; 15 *M*. *blythii* bats and three *M*. *schreibersii* bats were co-infected with *Bartonella* and *Leptospira*. Our results suggest that bats in Georgia are exposed to multiple bacterial infections. Further studies are needed to evaluate pathogenicity of these agents to bats and their zoonotic potential.

## Introduction

Bats (Chiroptera) represent one of the most successfully evolved mammalian groups on Earth for their unique characteristics, such as a long lifespan, the capability to fly long distances during foraging and particularly during seasonal migrations, the ability to inhabit a multitude of diverse ecological niches, and the colonial habitation. The role of bats in epidemiology of zoonotic diseases is very important as they frequently live in close proximity to humans and serve as reservoirs to different pathogens that include viruses, bacteria, fungi and parasites [1,2]. Many previous and ongoing research activities predominantly focused on viral agents in bats [3,4], but little is known about a presence of bacterial pathogens [5].

The bacterial genera *Bartonella*, *Brucella*, *Leptospira*, and *Yersinia* each consist of multiple species, some of which are zoonotic pathogens causing diseases in domestic or companion animals and in humans. *Bartonella* infections have been reported from a variety of animals occurring over a broad geographic distribution. Around 30 species have been described within the genus and the number is still increasing [6]. Controversy has been raised in several studies regarding host specificity of *Bartonella* [7–9]. Importantly, bats in the Northern Hemisphere have been implicated as a reservoir of *B*. *mayotimonensis* that was described from a human case of endocarditis in the USA [10,11], although the mechanism of transmission between bats and humans remains unresolved.

Brucellosis is an important zoonotic disease caused by bacteria of the genus *Brucella*. Domestic animals such as cattle, goats, sheep, pigs, camel, buffalo and dogs serve as reservoir hosts. Humans can be infected after contacting infectious animals or drinking raw milk [12]. Knowledge of *Brucella* ecology in wildlife is limited although several species were described in rodents, foxes, and marine mammals [13–17]. Except for an old report of anti-*Brucella* agglutinins in vampire bats (*Desmodus rotundus*) in Brazil [18], no other studies have reported *Brucella* infection in bats.

Leptospirosis is a bacterial zoonosis caused by *L*. *interrogans* and other pathogenic spirochetes of the genus *Leptospira*. Animals and humans acquire the infection through contact with water or soil contaminated with the urine of infected animals or by direct contact with these animals [19,20]. *Leptospira* spp. are distributed worldwide in rats and many other mammalian species [21]. Recently, leptospiral infections have been identified in bats from several countries [22–24].

Among infections caused by *Yersinia* species, plague (*Y*. *pestis)* causes the most notorious disease, infecting many mammalian species along with humans. Yersiniosis, occurring as an enteric disease in humans, is caused by *Y*. *pseudotuberculosis* and *Y*. *enterocolitica*, both of which have a broad distribution [25]. These bacteria have been frequently isolated from a variety of wild and domestic animals [26,27], but compared to *Y*. *pestis* their association with wildlife is not as well studied. Recently, bats have been reported to be infected with *Yersinia* [28,29].

The country of Georgia is located between the Greater Caucasus and Lesser Caucasus mountain ridges at the intersection of Europe and Asia. There are 109 mammalian species and many associated zoonotic agents in this region [30,31]. Recent studies conducted in this country have demonstrated the presence of diverse *Bartonella* species in wild rodents [32] and suggested the role of rat-associated *Bartonella* as a causative agent for a human illness [33]. Brucellosis is endemic in the area, with *B*. *abortus* and *B*. *melitensis* actively circulating in livestock and affecting local residents [34]. Leptospirosis is also broadly distributed in the country with increasing morbidity in recent years [35].

Bats are abundant in Georgia with at least 29 species identified [36]. However, information on bacterial infectious agents in bats from this region was absent. Understanding the prevalence and distribution of zoonotic pathogens in local bats would be significant from the veterinary and public health perspectives. In this study, we evaluated the presence and distribution of *Bartonella*, *Brucella*, *Leptospira*, and *Yersinia* in bats collected from eight colonies located in four regions of Georgia.

## Material and Methods

### Ethics statement

The work was performed in compliance with the protocol approved by the CDC Institutional Animal Care and Use Committee (protocol #2096FRAMULX-A3). Permits for the list of bat species sought and the number of animals per colony available for sampling were obtained from the Ministry of Environmental and Natural Resources Protection of Georgia.

### Study sites and bat tissues collection

**Fig 1:**
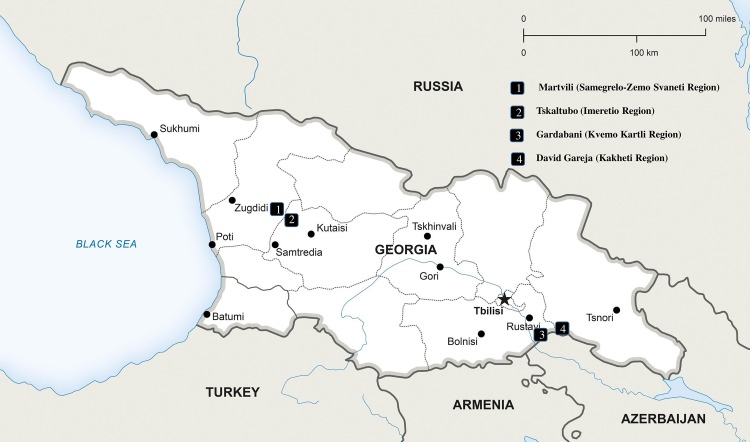
Bat sampling sites, Georgia, June 2012.

In June 2012, bats were captured manually or by using nets from eight colonies (found in caves, building attics, or monasteries) within four sites located in four regions of Georgia: one colony in Martvili (42°N, 42°E, Samegrelo-Zemo Svaneti region in western Georgia); three colonies in Tskaltubo (42°N, 42°E, Imereti region in west-central Georgia); one colony in Gardabani district (41°N, 45°E, Kvemo Kartli region in southern Georgia); and three colonies in David Gareja (41°N, 45°E, Kakheti region in eastern Georgia). Martvili and Tskaltubo, Gardabani and David Gareja, are neighboring sites, respectively (Fig 1).

Captured bats were delivered to the processing site in individual cotton bags. The bats were sexed, weighed, and identified to species based on external morphological characteristics. Bats were anesthetized using ketamine (0.05–0.1 mg/g body mass) and exsanguinated by cardiac puncture. Tissue samples (including spleen, kidney, intestine, and others) were collected from the bats. Samples were stored on dry ice in the field, then transferred to a -80°C freezer in the laboratory of Georgian NCDC before shipping to the US CDC’s laboratory in Fort Collins, Colorado for bacterial testing.

### DNA extraction and PCR detection

A small piece (~10 mg) of spleen, kidney, and intestine of each bat were homogenized separately using a Bullet Blender® Gold homogenizer (Next Advance, Averill Park, NY) following the protocols provided by the manufacturer. The homogenates were then transferred to a QIAxtractor (Qiagen, Valencia, CA) platform for DNA extraction using the tissue protocol, and the DNA was used as the template for downstream analyses. The kidney DNA was tested for *Bartonella* and *Leptospira*; the spleen DNA was tested for *Brucella* and *Yersinia*; and the intestine DNA was tested for *Yersinia* only. Molecular detection was performed using a conventional PCR assay carried out in a C1000 Touch Thermal Cycler (Bio-Rad, Hercules, CA) and/or real-time PCR assay carried out in a CFX96 Real-Time System (Bio-Rad, Hercules, CA). The relevant genes targeted were 16S – 23S internal transcribed spacer (ITS), insertion sequence (IS711), 32-kDa lipoprotein (*lipL32*) gene, and peptidoglycan-associated lipoprotein (*pal*) gene for *Bartonella*, *Brucella*, *Leptospira*, and *Yersinia*, respectively. The reactions for ITS were run under conventional PCR settings; the reactions for IS711, *pal* and *lipL32*were run under the real-time multiplex PCR settings, following protocols published elsewhere [37–39]. Ct value < 36 with an amplification curve is recorded as positive. Positive samples for *Brucella* DNA (IS711) and *Leptospira* DNA (*lipL32*) identified by real-time PCR were further tested by conventional PCR targeting a 223 base pair-fragment in 31 kDa gene (*bcsp31*) for *Brucella* [40] and a 423 base pair-fragment in *lipL32* for *Leptospira* using primers [41] different from the real-time PCR. All primers and probes used in this study are listed in Table 1. The *pal* primers and probes for detection of *Yersinia* species were developed for this study based on a whole-genome scan (M. Diaz, unpublished data). For any conventional PCR, the PCR products were analyzed for the presence of amplicons of the expected size by electrophoresis on 1.5% agarose gels containing GelGreen stain (Biotium, Hayward, CA). Positive and negative controls were included in each PCR assay to evaluate the presence of appropriately sized amplicons and to rule out potential contamination, respectively.

**Table 1:** Molecular detection of bacterial agents in bats from Georgia, June 2012.

Agents	Gene target	PCR assay	Primer/probe sequences	Reference
*Bartonella*	ITS	conventional	Forward: CTT CAG ATG ATG ATC CCA AGC CTT CTG GCG	[39]
			Reverse: GAA CCG ACG ACC CCC TGC TTG CAA AGC A	
			Forward: GCT TGA AGC TTG CGG ACA GT	
*Brucella*	*IS711*	real-time	Reverse: GGC CTA CCG CTG CGA AT	[37]
			Probe: AAG CCA ACA CCC GGC CAT TAT GGT	
*Brucella*	*bcsp31*	conventional	Forward: TGG CTC GGT TGC CAA TAT CAA	[40]
			Reverse: CGC GCT TGC CTT TCA GGT CTG	
			Forward: AAG CAT TAC CGC TTG TGG TG	
*Leptospira*	*lipL32*	real-time	Reverse: GAA CTC CCA TTT CAG CGA TT	[38]
			Probe: AA AGC CAG GAC AAG CGC CG	
*Leptospira*	*lipL32*	conventional	Forward: CGC TGA AAT GGG AGT TCG TAT GAT T	[41]
			Reverse: CCA ACA GAT GCA ACG AAA GAT CCT TT	
			Forward: CGC AAA TAA TGA CCA ATC TGG	
*Yersinia*	*Pal*	real-time	Reverse: CGT GGC CTT CAA CAA CAA C	This study
* *	* *		Probe: CGG TTC TGA CTT CGC TCA AAT GCT GG	

*Leptospira* infection rate was compared between study sites and between bat species using chi-square tests.

### Sequencing and phylogenetic analysis for *Bartonella* species and *Leptospira* species

Samples positive for *Bartonella* (ITS) and *Leptospira* (*lipL*32) were further identified by sequencing analyses. The PCR amplicons were purified using a QIAquick PCR Purification Kit (Qiagen, Valencia, CA) according to manufacturer’s instructions, and then sequenced in both directions using ABI 3130 Genetic Analyzer (Applied Biosystems, Foster City, CA). Forward and reverse sequences were assembled using the SeqMan Pro program in Lasergene v.12 (DNASTAR, Madison, WI). Assembled sequences obtained from all samples in the present study were compared between themselves and with reference sequences available in GenBank after alignment using the Clustal algorithm in the MegAlign program in Lasergene. Using the neighbor-joining method, cladogram (showing the cladistics relationship rather than phylogenetic relationship) was generated for *Bartonella* among the ITS sequences. Sequences were assigned to clades visually based on monophyletic clusters. Phylogenetic tree was constructed for *Leptospira*, Branch support was estimated using 1000 bootstrap replicates. Newly identified sequence variants were submitted to GenBank.

## Results

### Bat sampling

A total of 236 bats were captured from the trapping sites. Samples with incomplete or missing information were excluded, which resulted in 218 bats available for analysis. The animals belonged to eight species of five genera, including *Eptesicus serotinus* (n = 17), *Miniopterus schreibersii* (n = 27), *Myotis blythii* (n = 68), *Myotis emarginatus* (n = 42), *Myotis mystacinus* (n = 1), *Pipistrellus pygmaeus* (n = 11), *Rhinolophus euryale* (n = 28), and *R*. *ferrimequinum* (n = 24) (Table 2). The *Myotis* spp. bats accounted for more than half of the tested bats. Other bat species accounted for a smaller portion, ranging from 5% to 13%.

**Table 2:** Detection of *Bartonella*, *Brucella*, and *Leoptospira* in bats from Georiga, 2012.

Bat species	# Tested	*Bartonella*		*Brucella*		*Leptospira*		*Yersinia*	
		# Pos	Prevalence (%)	# Pos	Prevalence (%)	# Pos	Prevalence (%)	# Pos	Prevalence (%)
*Eptesicus serotinus*	17	1	6	0	0	0	0	0	0
*Miniopterus schreibersii*	27	13	48	2	7	4	15	0	0
*Myotis blythii*	68	26	38	2	3	21	31	0	0
*Myotis emarginatus*	42	12	29	0	0	0	0	0	0
*Myotis mystacinus*	1	0	0	0	0	0	0	0	0
*Pipistrellus pygmaeus*	11	1	9	0	0	0	0	0	0
*Rhinolophus euryale*	28	12	43	0	0	0	0	0	0
*Rhinolophus ferrimequinum*	24	12	50	0	0	0	0	0	0
Total	218	77	35	4	2	25	11	0	0

The number of bat species varied by site, with three, four, five, and three in Martvili, Tskaltubo, Gardabani, and David Gareja, respectively. All *M*. *schreibersii* bats (n = 27) were collected in Tskaltubo. *M*. *blythii* bats were mainly captured in Tskaltubo and David Gareja (Table 3).

**Table 3:** Geographic distribution of the captured bats, Georgia, June 2012.

Bat species	Martvili	Tskaltubo	Gardabani	David Gareja	Total
*Eptesicus serotinus*			17		17
*Miniopterus schreibersii*		27			27
*Myotis blythii*	2	44		22	68
*Myotis emarginatus*		14	15	13	42
*Myotis mystacinus*			1		1
*Pipistrellus pygmaeus*			11		11
*Rhinolophus euryale*	13	15			28
*Rhinolophus ferrimequinum*	5		1	18	24
Total	20	100	45	53	218

### Molecular detection

*Bartonella* DNA was detected in 77 of the 218 tested bat kidneys, giving the overall prevalence of 35%. *Bartonella* DNA was detected in all bat species but *M*. *mystacinus*, for which there was only one sample available for testing. The prevalence of *Bartonella* infection ranged from 6% in *E*. *serotinus* to 50% in *R*. *ferrimequinum* (Table 2). The *Bartonella-*positive bats were present in all colonies, with variable numbers of 4–23 per colony (the proportion of infected bats per colony cannot be estimated as the size of the colonies was not evaluated).

*Brucella* DNA was detected in spleen samples of four bats by real-time PCR (IS711) and confirmed by conventional PCR (*bcsp31*). The positive batsinclude two *M*. *schreibersii* and two *M*. *blythii*. All of the four *Brucella-*positive bats were obtained from one site (Tskaltubo) with three of them from one colony and the last one (*M*. *schreibersii*) from a neighboring colony.

*Leptospira* DNA was amplified from kidneys of 25 bats by real-time PCR (*lipL32*) and confirmed by conventional PCR (*lipL32*). The positive bats were *M*. *schreibersii* (n = 4) or *M*. *blythii* (n = 21). The prevalence in *M*. *blythii* (31%; 21/68) was significantly higher (χ^2^ = 1.89, p <0.05) than that in *M*. *schreibersii* (15%; 4/27). The *Leptospira-*positive bats were found in four colonies within two sites–one colony in David Gareja and the other three colonies in Tskaltubo. All four positive *M*. *schreibersii* were collected in Tskaltubo; the *M*. *blythii* were distributed in David Gareja (n = 8) and Tskaltubo (n = 13). The infection rate of *Leptospira* in *M*. *blythii* was 36% (8/22) in David Gareja and 30% (13/44) in Tskaltubo, with no statistical difference observed between the two sites (χ^2^ = 0.20, p >0.05).

*Yersinia* DNA was detected in none of the bats, neither in spleen nor intestine.

### Mixed infection

Two or three pathogens were detected in *M*. *blythii* and *M*. *schreibersii*. Two bats (one *M*. *blythii* and one *M*. *schreibersii*) captured in different colonies within site Tskaltubo were co-infected with all three pathogens–*Bartonella*, *Brucella*, and *Leptospira*. Two bats (one *M*. *blythii* and one *M*. *schreibersii*) captured in the same colony within site Tskaltubo were co-infected with *Bartonella* and *Brucella*; and eighteen bats (15 *M*. *blythii* and three *M*. *schreibersii*) captured in one colony in site David Gareja and in two colonies in site Tskaltubo were co-infected with *Bartonella* and *Leptospira*. No mixed infections were observed in the remaining bat species.

### Sequencing analysis

Sequencing analyses were performed on ITS sequences of *Bartonella* and *lipL*32 sequences of *Leptospira*, both of which were amplified from bat kidney DNA.

**Fig 2:**
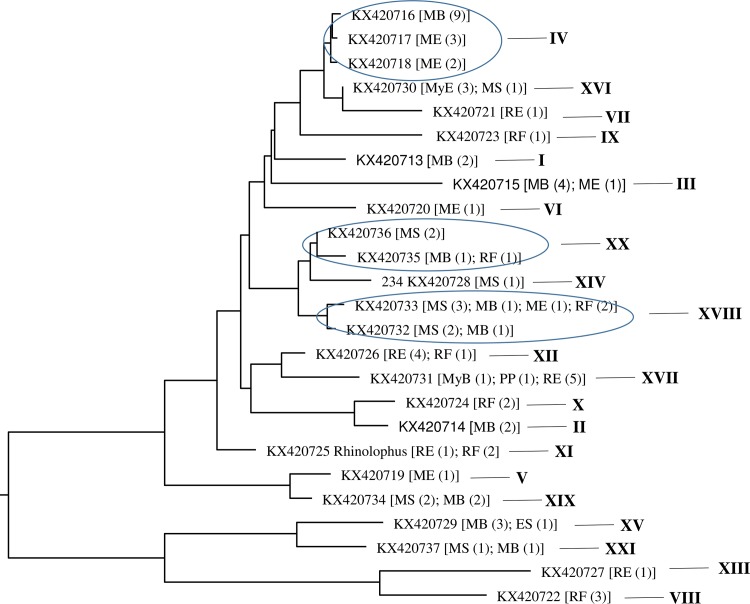
Cladistics relationship of the *Bartonella* variants detected in bats from Georgia based on ITS sequences. A total of 77 *Bartonella* ITS sequences were obtained. The sequences belonged to 25 variants (each indicated by a unique GenBank accession number), and the variants clustered into 21 clades (marked by a unique Roman number). After each variant, it is host species and number of *Bartonella* sequences obtained from the host species. The cladogram was generated by neighboring-joining method. ES: *Eptesicus serotinus*; MB: *Myotis blythii*; ME: *Myotis emarginatus*; MS: *Miniopterus schreibersii*; PP: *Pipistrellus pygmaeus*; RE: *Rhinolophus euryale*; RF: *Rhinolophus ferrimequinum*.

The ITS sequences of *Bartonella* exhibited considerable heterogeneity. The 77 *Bartonella*-positive DNA represented 25 genetic variants (a variant is defined when at least one nucleotide difference is observed between compared sequences). These variants were all unique and submitted to GenBank (accession numbers KX420713—KX420737). Only a few variants were relatively close (96.3% similarity) between themselves, while the majority were largely distant from each other and clustered into 21 clades (a clade is a cluster of sequences following neighbor-joining analysis) (Fig 2). Except for *E*. *serotinus* and *P*. *pygmaeus* in which *Bartonella* was detected in one individual for each species, each of the other bat species was associated with 4 to 9 *Bartonella* clades. For example, *M*. *blythii* was associated with nine lineages (I—IV, XV, and XVII—XXI); *M*. *schreibersii* was associated with six lineages (III—VI, XVI, and XVIII); *R*. *euryale* was associated with eight lineages (VII—XIII and XVII). Most *Bartonella* lineages were specific to certain bat genus/species. In particular, lineages I—VI and lineages VII—XIII were associated with *Myotis* spp. and *Rhinolophus* spp., respectively; while lineages XV—XXI could be associated with multiple bat genera (Fig 2).

**Fig 3:**
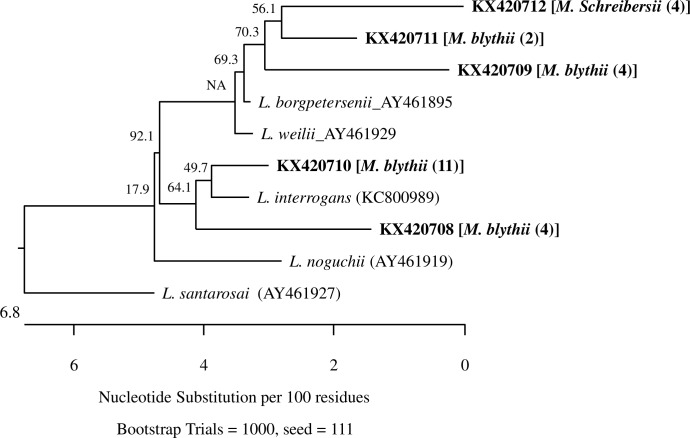
Phylogenetic relationships of the five *Leptospira* variants detected in bats from Georgia based on *lipL32* sequences. Each variant is indicated by a unique GenBank accession number, and followed by bat species name and sequences obtained from the bat species. Phylogenetic tree was constructed by neighboring-joining method, and bootstrap values were calculated with 1,000 replicates.

The 25 *LipL*32 sequences of *Leptospira* detected in the bats represented five variants with sequence distances of 3.7% - 7.3% (Fig 3). All variants were novel and assigned GenBank accession numbers KX420708 –KX420712. Of the 25 sequences, the four sequences recovered from *M*. *schreibersii* were identical (variant KX420712); while the other 21 sequences from *M*. *blythii* were of four variants, each of which was detected in 11, 4, 2, and 4 individuals, respectively. These variants were close to some *Leptospira* species that are known zoonotic pathogens. Specifically, variant KX420710 that was detected in 11 *M*. *blythii* was closest to *L*. *interrogans* with genetic identity of 98.6%; while a few other variants were relatively close to *L*. *borgpetersenii* with genetic identity of 96% - 97%.

## Discussion

Using molecular approaches, we report the detection of multiple potential zoonotic pathogens, including *Bartonella*, *Brucella*, and *Leptospira* in bats from the country of Georgia.

Similar to early reports from other parts of the world [8,42], *Bartonella* species are widely distributed in bats in Georgia with very high diversity. All genetic variants discovered in the study were novel and do not belong to any previously described *Bartonella* species. Further characterization is necessary to verify whether the identified DNA sequences represent novel *Bartonella* species. Most *Bartonella* variants described here show specific relationships to their bat hosts at a genus level, particularly *Myotis* spp. and *Rhinolophus* spp. Conversely, some *Bartonella* variants were shared among bats of different genera, which suggests frequent host-shifting or cross-species transmission potentially related to exchange of ectoparasites between bats [43,44]. We do not know whether the *Bartonella* variants identified in the bats are responsible for any human disease in Georgia, but a recent report that bats in northern Europe harbor a human pathogen *B*. *mayotimonensis* [11] indicates such a possibility. New information about *Bartonella* infection in bats in Georgia can provide additional insights towards understanding the interactions between humans, animals, and parasites.

*Leptospira* infection was detected in the tested bats. Interestingly, all *Leptospira* infected-bats were either *M*. *blythii* or *M*. *schreibersii*. No *Leptospira* infection was detected in bats of other species, including the *M*. *emarginatus* which is a quite common local species. Species-specific variations in bacterial infection rates may indicate that certain bat species are more exposed habitually (e.g. drinking the same contaminated water) or even more susceptible to *Leptospira* than other bat species [23,45,46]. Our observation of similar *Leptospira* infection rates in *M*. *blythii* in Tskaltubo and David Gareja suggests that these bats were equally exposed to the infection at different sites; while higher prevalence in *M*. *blythii* than in *M*. *schreibersii* may suggest that *M*. *blythii* is more susceptible to *Leptospira*. Field observations showed that *M*. *blythii* and *M*. *schreibersii* share the same roosts and *M*. *schreibersii* usually incorporate into the dense groups of *M*. *blythii*, which presumably results in close body to body contact between animals of these two species. *M*. *blythii* may transmit the infection to *M*. *schreibersii* through urinary shedding and other similar routes. The high prevalence observed suggests that these bats might play a possible role in the maintenance of *Leptospira* spp. in the environment [47]. On the other hand, one *Leptospira* variant identified in *M*. *blythii* was closely related to *L*. *interrogans* (98.6% identity), a well-known zoonotic pathogen frequently found in rats [48]. Considering the high frequency of this variant (detected in 11 of 25 or 44% infected bats), it warns that *M*. *blythii* may serve as a natural reservoir to *L*. *interrogans* and can potentially transmit the infection to humans, particularly when they roost synantropically, e.g. in monasteries. Furthermore, some of the variants were relatively close to *L*. *borgpetersenii* that also is a zoonotic pathogen.

The most intriguing finding of this work is probably the discovery of *Brucella* in bats. Although there was a single report of *Brucella* agglutinins in *Desmodus rotundus* bats from Brazil [18], our study represents the first detection of *Brucella* DNA in bats. Similar to the finding of *Leptospira* spp., the *Brucella* infections were found only in *M*. *schreibersii* and *M*. *blythii*. Interestingly, all *Brucella* positive bats were from the site Tskaltubo. The habitat preference and geographic origin could influence the infection prevalence in these bats. It was evident for *M*. *schreibersii* since this species was only captured in Tskaltubo; whereas absence of *Brucella*-positive *M*. *blythii* bats in David Gareja (where no *M*. *schreibersii* were present) may suggest that *M*. *blythii* contracted *Brucella* infection from *M*. *schreibersii* in Tskaltubo. Alternatively, as was the case with *Leptospira*, bats of both species in Tskaltubo might be exposed to *Brucella* independently by consuming the same contaminated source, such as water. The most studied *Brucella* species (*B*. *melitensis*, *B*. *abortus*, and *B*. *suis*) are so genetically similar that some researchers have argued for their combination into one species [49]. However, the epidemiological and diagnostic benefits for separating the genus based on phenotypic and ecological characteristics are more compelling. Therefore, it is important to distinguish genotypic and phenotypic differences between this newly discovered bat-associated *Brucella* and other *Brucella* species. In the current presentation, we only report the detection of *Brucella* DNA in the bats. Further characterization of the bacterium is in progress. Because most *Brucella* species are highly pathogenic, it is important to determine whether this strain has a zoonotic potential.

We did not find any *Yersinia* infections in this study using recently designed primers. Since the sensitivity of the utilized primers for detection of all *Yersinia* species in the field samples has not been sufficiently evaluated in other field studies, the negative results for this bacterial genus in Georgian bats might represent a lack of sensitivity of our assay or a need for more broadly reactive primers.

In conclusion, bats from Georgia harbor several potential bacterial pathogens. These preliminary data highlight that bats may play an important role in maintaining those agents in nature. Further studies need to be carried out to understand the importance of these agents for bats, other wildlife, veterinary and public health.

## References

[1] Whitaker JO Jr, Ritzi CM, Dick CW. Collecting and preserving ectoparasites for ecological study In: Kunz TH, Parsons S (eds) Ecological and Behavioral Methods for the Study of Bats. 2009; 2nd edn: 806–827. The Johns Hopkins University Press, Baltimore, MD.

[2] Wibbelt G, Speck S, Field H. Methods for assessing diseases in bats In: Kunz TH, Parsons S (eds) Ecological and Behavioral Methods for the Study of Bats. 2009; 2nd edn: 775–794. The Johns Hopkins University Press, Baltimore, MD.

[3] Messenger SL, Smith JS, Orciari LA, Yager PA, Rupprecht CE. Emerging pattern of rabies deaths and increased viral infectivity. Emerg Infect Dis. 2003;9(2):151–4. 10.3201/eid0902.020083 12603983 PMC2901935

[4] Calisher CH, Childs JE, Field HE, Holmes KV, Schountz T. Bats: important reservoir hosts of emerging viruses. Clin Microbiol Rev. 2006;19(3):531–45. 10.1128/CMR.00017-06 16847084 PMC1539106

[5] Mühldorfer K. Bats and bacterial pathogens: a review. Zoonoses Public Health. 2013;60(1):93–103. 10.1111/j.1863-2378.2012.01536.x 22862791

[6] Carrasco SE, Chomel BB, Gill VA, Kasten RW, Maggi RG, Breitschwerdt EB, et al Novel *Bartonella* infection in northern and southern sea otters (*Enhydra lutris kenyoni* and *Enhydra lutris nereis*). Vet Microbiol. 2014;170(3–4):325–34. 10.1016/j.vetmic.2014.02.021 24629902

[7] Bai Y, Calisher C, Kosoy M, Root J, Doty J. Persistent infection or successive reinfection of deer mice with *Bartonella vinsonii* subsp. *arupensis*. Appl Environ Microbiol. 2011;77(5):1728–31. 10.1128/AEM.02203-10 21239553 PMC3067298

[8] Bai Y, Kosoy M, Recuenco S, Alvarez D, Moran D, Turmelle A, et al *Bartonella* spp. in bats, Guatemala. Emerg Infect Dis. 2011;17(7):1269–72. 10.3201/eid1707.101867 21762584 PMC3381397

[9] Bai Y, Malania L, Castillo DA, Moran D, Boonmar S, Chanlun A, et al Global distribution of *Bartonella* infections in domestic bovine and characterization of *Bartonella bovis* strains using multi-locus sequence typing. PLoS One. 2013;8(11):e80894 10.1371/journal.pone.0080894 24278342 PMC3836770

[10] Lin EY, Tsigrelis C, Baddour LM, Lepidi H, Rolain JM et al Candidatus *Bartonella mayotimonensis* and endocarditis. Emerg Infect Dis. 2010;16(3):500–3. 10.3201/eid1603.081673 20202430 PMC3321999

[11] Veikkolainen V, Vesterinen EJ, Lilley TM, Pulliainen AT. Bats as reservoir hosts of human bacterial pathogen, *Bartonella mayotimonensis*. Emerg Infect Dis. 2014;20(6):960–7. 10.3201/eid2006.130956 24856523 PMC4036794

[12] Haque N, Bari MS, Hossain MA, Muhammad N, Ahmed S, Rahman A, et al An overview of Brucellosis. Mymensingh Med J. 2011;20(4):742–7. 22081201

[13] Stoenner HG, Lackman DB. A new species of *Brucella* isolated from the desert wood rat, *Neotoma lepida* Thomas. Am J Vet Res. 1957;18(69):947–51. 13470254

[14] Ewalt DR, Payeur JB, Martin BM, Cummins DR, Miller WG. Characteristics of a *Brucella* species from a bottlenose dolphin (*Tursiops truncates*). J Vet Diagn Invest. 1994;6(4):448–52. 7858024 10.1177/104063879400600408

[15] Hubálek Z, Scholz HC, Sedlácek I, Melzer F, Sanogo YO, Nesvadbová J. Brucellosis of the common vole (*Microtus arvalis*). Vector Borne Zoonotic Dis. 2007;7(4):679–87. 10.1089/vbz.2007.0143 18021023

[16] Scholz HC, Hofer E, Vergnaud G, Le Fleche P, Whatmore AM, Al Dahouk S. Isolation of *Brucella microti* from mandibular lymph nodes of red foxes, *Vulpes vulpes*, in lower Austria. Vector Borne Zoonotic Dis. 2009;9(2):153–6. 10.1089/vbz.2008.0036 18973444

[17] Tiller RV, Gee JE, Frace MA, Taylor TK, Setubal JC, Hoffmaster AR, et al Characterization of novel *Brucella* strains originating from wild native rodent species in North Queensland, Australia. Appl Environ Microbiol. 2010;76(17):5837–45. 10.1128/AEM.00620-10 20639360 PMC2935067

[18] Ricciardi ID, Nunes MP, Andrade CM, Da Silva AG. Anti-brucella agglutinins in bats and "Callithrix" monkeys. J Wildl Dis. 1976;12(1):52–4. 815569 10.7589/0090-3558-12.1.52

[19] Levett PN. Leptospirosis. Clin Microbiol Rev. 2001;14(2):296–326. 10.1128/CMR.14.2.296-326.2001 11292640 PMC88975

[20] Adler B, Moctezuma AP. Leptospira and leptospirosis. Vet Microbiol. 2010;140(3–4):287–96. 10.1016/j.vetmic.2009.03.012 19345023

[21] Acha PN, Szyfres B. Zoonosis y enfermedades transmisibles comunes al hombre y a los animales (3. ed.), 1, Organización Panamericana de la Salud, Washington. 2001;pp. 175–186 (Publicación Científica y Técnica, 580).

[22] Cox TE, Smythe LD, Leung LK. Flying foxes as carriers of pathogenic *Leptospira* species. J Wildl Dis. 2005;41(4):753–7. 10.7589/0090-3558-41.4.753 16456164

[23] Matthias MA, Díaz MM, Campos KJ, Calderon M, Willig MR, Pacheco V, et al Diversity of bat-associated *Leptospira* in the Peruvian Amazon inferred by bayesian phylogenetic analysis of 16S ribosomal DNA sequences. Am J Trop Med Hyg. 2005;73(5):964–74. 16282313 PMC2270400

[24] Dietrich M, Mühldorfer K, Tortosa P, Markotter W (2015) Leptospira and Bats: Story of an Emerging Friendship. PLoS Pathog. 2015;11(11):e1005176 10.1371/journal.ppat.1005176 26562435 PMC4643053

[25] Dollinger P, Baumgartner R, Hatt JM, Isenbugel E, Pagan O. Zoonoses surveillance and safeguard measures in Swiss zoos. Proc EAZWV and BVSII Scientific Meeting, Chester, United Kingdom. 1998;1–12.

[26] Mair NS. Pseudotuberculosis in free-living wild animals. Symp Zool Soc Lond. 1968;24: 107–117.

[27] Mair NS. Yersiniosis in wildlife and its public health implications. Wildl Dis. 1973;9(1):64–71. 10.7589/0090-3558-9.1.64 4571715

[28] Childs-Sanford SE, Kollias GV, Abou-Madi N, McDonough PL, Garner MM, Mohammed HO. *Yersinia pseudotuberculosis* in a closed colony of Egyptian fruit bats (*Rousettus aegyptiacus*). J Zoo Wildl Med. 2009;40(1):8–14. 10.1638/2007-0033.1 19368235

[29] Mühldorfer K, Wibbelt G, Haensel J, Riehm J, Speck S. Yersinia species isolated from bats, Germany. Emerg Infect Dis. 2010;16(3):578–80. 10.3201/eid1603.091035 20202457 PMC3322022

[30] Bondyrev IV, Davitashvili ZV, Singh VP. The geography of Georgia—problems and perspectives. 2015;228pp. Springer.

[31] Han BA, Kramer AM, Drake JM. Global Patterns of zoonotic disease in mammals. Trends Parasitol. 2016 7;32(7):565–77. 10.1016/j.pt.2016.04.007 27316904 PMC4921293

[32] Malania L, Bai Y, Osikowicz LM, Tsertsvadze N, Katsitadze G, Imnadze P, et al Prevalence and diversity of *Bartonella* species in rodents from Georgia (Caucasus). Am J Trop Med Hyg. 2016;95(2):466–71. 10.4269/ajtmh.16-0041 27162268 PMC4973202

[33] Kandelaki G, Malania L, Bai Y, Chakvetadze N, Katsitadze G, Imnadze P, et al (2016) Human lymphadenopathy caused by ratborne *Bartonella*, Tbilisi, Georgia. Emerg Infect Dis. 2016;22(3):544–6. 10.3201/eid2203.151823 26889959 PMC4766887

[34] Sanodze L, Bautista CT, Garuchava N, Chubinidze S, Tsertsvadze E, Broladze M, et al Expansion of brucellosis detection in the country of Georgia by screening household members of cases and neighboring community members. BMC Pubic Health. 2015;15:459. 10.1186/s12889-015-1761-y PMC4432945 25934639

[35] Mamuchishvili N, Kuchuloria T, Mchedlishvili I, Imnadze P. Leptospirosis in Georgia. Georgian Med News. 2014;(228):63–6. 24743125

[36] Yavruyan E, Rakhmatulina I, Bukhnikashvili A, Kandaurov A, Natradze I, Gazaryan S. Bats conservation action plan for the Caucasus. 2008;83pp. Universal, Tbilisi, Georgia.

[37] Halling SM, Tatum FM, Bricker BJ. Sequence and characterization of an insertion sequence, IS711, from *Brucella ovis*. Gene. 1993;133(1):123–7. 8224885 10.1016/0378-1119(93)90236-v

[38] Stoddard RA. Detection of pathogenic *Leptospira* spp. through real-time PCR (qPCR) targeting the *LipL32* gene. Methods Mol Biol. 2013;943:257–66. 10.1007/978-1-60327-353-4_17 23104295

[39] Diniz PP, Maggi RG, Schwartz DS, Cadenas MB, Bradley JM, Hegarty B, et al Canine bartonellosis: serological and molecular prevalence in Brazil and evidence of co-infection with *Bartonella henselae* and *Bartonella vinsonii* subsp. *berkhoffii*. Vet Res. 2007;38(5):697–710. 10.1051/vetres:2007023 17583666

[40] Baily GG, Krahn JB, Drasar BS, Stoker NG. Detection of *Brucella melitensis* and *Brucella abortus* by DNA amplification. J Trop Med Hyg. 1992;95(4):271–5. 1495123

[41] Levett PN, Morey RE, Galloway RL, Turner DE, Steigerwalt AG, Mayer LW. Detection of pathogenic leptospires by real-time quantitative PCR. J Med Microbiol. 2005;54(Pt 1):45–9. 10.1099/jmm.0.45860-0 15591254

[42] Kosoy M, Bai Y, Lynch T, Kuzmin IV, Niezgoda M, Franka R, et al Bartonella spp. in bats, Kenya. Emerg Infect Dis. 2010;16(12):1875–81. 10.3201/eid1612.100601 21122216 PMC3294596

[43] Morse SF, Olival KJ, Kosoy M, Billeter S, Patterson BD, Dick CW, et al Global distribution and genetic diversity of Bartonella in bat flies (Hippoboscoidea, Streblidae, Nycteribiidae). Infect Genet Evol. 2012;12(8):1717–23. 10.1016/j.meegid.2012.06.009 22771358

[44] McKee CD, Hayman DT, Kosoy MY, Webb CT (2016) Phylogenetic and geographic patterns of bartonella host shifts among bat species. Infect Genet Evol. 2016;44:382–94. 10.1016/j.meegid.2016.07.033 27473781 PMC5025394

[45] Fennestad KL, Borg-Petersen C (1972) Leptospirosis in Danish wild mammals. J Wildl Dis. 1972;8(4):343–51. 4564183 10.7589/0090-3558-8.4.343

[46] Bessa TA, Spichler A, Chapola EG, Husch AC, de Almeida MF, Sodré MM, et al The contribution of bats to leptospirosis transmission in Sa˜o Paulo City, Brazil. Am J Trop Med Hyg. 2010;82(2):315–7. 10.4269/ajtmh.2010.09-0227 20134010 PMC2813174

[47] Everard CO, Fraser-Chanpong GM, Bhagwandin LJ, Race MW, James AC. Leptospires in wildlife from Trinidad and Grenada. J Wildl Dis. 1983;19(3):192–9. 6644917 10.7589/0090-3558-19.3.192

[48] Cosson JF, Picardeau M, Mielcarek M, Tatard C, Chaval Y, Suputtamongkol Y, et al Epidemiology of *Leptospira* transmitted by rodents in Southeast Asia. PLoS Negl Trop Dis. 2014;8(6):e2902 10.1371/journal.pntd.0002902 24901706 PMC4046967

[49] Olsen SC, Palmer MV (2014) Advancement of knowledge of Brucella over the past 50 years. Vet Pathol. 2014;51(6):1076–89. 10.1177/0300985814540545 24981716

